# Alpha Lipoic Acid Efficacy in PCOS Treatment: What Is the Truth?

**DOI:** 10.3390/nu15143209

**Published:** 2023-07-19

**Authors:** Alice Guarano, Anna Capozzi, Martina Cristodoro, Nicoletta Di Simone, Stefano Lello

**Affiliations:** 1 Department of Biomedical Sciences, Humanitas University, Via Rita Levi Montalcini 4, 20072 Pieve Emanuele, Milan, Italy; alice.guarano@sanpiox.humanitas.it (A.G.); martina.cristodoro@st.hunimed.eu (M.C.); 2 Humanitas San Pio X, Via Francesco Nava 31, 20159 Milan, Italy; 3 Dipartimento di Scienze della Salute della Donna, del Bambino e di Sanità Pubblica, Fondazione Policlinico Universitario Agostino Gemelli Istituto di Ricovero e Cura a Carattere Scientifico (IRCCS), Largo Agostino Gemelli 8, 00168 Rome, Italy; anna.capozzi@guest.policlinicogemelli.it (A.C.); lello.stefano@gmail.com (S.L.); 4 IRCCS Humanitas Research Hospital, Via Manzoni 56, 20089 Rozzano, Milan, Italy

**Keywords:** alpha lipoic acid, polycystic ovary syndrome, insulin resistance, chronic inflammation, insulin-sensitizing factors

## Abstract

Polycystic ovary syndrome (PCOS) is among the most common female endocrinopathies, affecting about 4–25% of women of reproductive age. Women affected by PCOS have an increased risk of developing metabolic syndrome, type 2 diabetes mellitus, cardiovascular diseases, and endometrial cancer. Given the pivotal role of insulin resistance (IR) in the pathogenesis of PCOS, in the last years, many insulin-sensitizing factors have been proposed for PCOS treatment. The first insulin sensitizer recommended by evidence-based guidelines for the assessment and treatment of PCOS was metformin, but the burden of side effects is responsible for treatment discontinuation in many patients. Inositols have insulin-mimetic properties and contribute to decreasing postprandial blood glucose, acting by different pathways. ALA is a natural amphipathic compound with a very strong anti-inflammatory and antioxidant effect and a very noteworthy role in the improvement of insulin metabolic pathway. Given the multiple effects of ALA, a therapeutic strategy based on the synergy between inositols and ALA has been recently proposed by many groups with the aim of improving insulin resistance, reducing androgen levels, and ameliorating reproductive outcomes in PCOS patients. The purpose of this study is to review the existing literature and to evaluate the existing data showing the efficacy and the limitation of a treatment strategy based on this promising molecule. ALA is a valid therapeutic strategy applicable in the treatment of PCOS patients: Its multiple actions, including antinflammatory, antioxidant, and insulin-sensitizing, may be of utmost importance in the treatment of a very complex syndrome. Specifically, the combination of MYO plus ALA creates a synergistic effect that improves insulin resistance in PCOS patients, especially in obese/overweight patients with T2DM familiarity. Moreover, ALA treatment also exerts beneficial effects on endocrine patterns, especially if combined with MYO, improving menstrual regularity and ovulation rhythm. The purpose of our study is to review the existing literature and to evaluate the data showing the efficacy and the limitations of a treatment strategy based on this promising molecule.

## 1. Introduction

Polycystic ovary syndrome (PCOS) is among the most common female endocrinopathies, affecting about 4–25% of women of reproductive age, with different levels of prevalence according to diagnostic criteria and analyzed populations [1]. Most of the international societies guidelines endorse Rotterdam criteria for the diagnosis of PCOS [2,3]. According to Rotterdam criteria, PCOS is diagnosed when at least two of the following conditions are fulfilled, and other conditions which may interfere with these signs and symptoms are ruled out: (1) irregular menstrual cycles and/or ovulatory dysfunctions (oligo or anovulation), (2) clinical or biochemical hyperandrogenism (hirsutism, alopecia, and/or acne, elevated calculated free testosterone, free androgen index, or calculated bioavailable testosterone), (3) polycystic morphology of the ovaries at ultrasound scan (a follicle number per ovary >20 and/or an ovarian volume >/= 10 mL) [2,4,5]. PCOS is a complex syndrome that reflects multiple potential etiologies: it probably has a multifactorial origin, with an individual susceptibility determined by various risk factors, both genetic and environmental, such as diet and lifestyle.

Because of its multifactorial nature, PCOS’s etiology is still poorly understood, but lately, the attention has been set on some risk factors [6,7,8]: insulin resistance, chronic inflammation, and consequentially, oxidative stress [9] may play an essential role in the pathogenesis of PCOS.

PCOS has a strong impact on endocrine metabolic system. At least 50% of women affected by this syndrome are obese [2], many have insulin resistance [3], dyslipidemia [10], and a low-grade chronic inflammation state, which can lead to a systemic endothelial dysfunction [11,12,13]. Therefore, women affected by PCOS have an increased risk of metabolic syndrome, type 2 diabetes mellitus, cardiovascular diseases, and endometrial cancer.

Additionally, PCOS may interfere with reproduction. In fact, it represents one of the most common causes of infertility in women of reproductive age because of the possible impact of hormonal disturbances on ovulation and conception. Moreover, the low-grade inflammation associated with the syndrome may negatively influence oocyte quality.

PCOS patients are also at risk of developing obstetric conditions, such as recurrent pregnancy loss, gestational diabetes, and psychological issues, such as anxiety, depressive symptoms, psychosexual dysfunction, and eating disorders [1,2]. 

Therefore, early identification of this disease is essential to better establish the appropriate therapeutic strategies, both pharmacological and non-pharmacological.

## 2. Treatment Strategies of PCOS

Given the pivotal role of insulin resistance (IR) in the pathogenesis of PCOS, in the last years, many insulin-sensitizing factors have been proposed for PCOS treatment [14,15].

Metformin is recommended by the 2018 international evidence-based guidelines for the assessment and treatment of PCOS [1] for the management of weight, hormonal, and metabolic outcomes in PCOS adult patients, where lifestyle modifications are not sufficient. Metformin is a biguanide and it has been reported to ameliorate the glucose–insulin metabolism by reducing intestinal absorption of glucose, glycogenolysis, gluconeogenesis, and lipogenesis and by stimulating the uptake of glucose in liver, adipose tissue, skeletal muscle, and ovaries. In a meta-analysis including seven randomized controlled trials (RCTs) that evaluated the comparison between lifestyle vs. metformin + lifestyle, no statistically significant differences were found for weight management and BMI, but when administered alone, it resulted superior to placebo for the most important clinical outcomes [16]. Regarding the reproductive outcomes, metformin could be administered alone or in combination with estroprogestins, ovulation induction agents, or during in vitro fertilization (IVF) programs. A Cochrane review including a meta-analysis of 42 randomized clinical trials reported how metformin therapy improved menstrual pattern, ovulation rate, and clinical pregnancy rate in PCOS women [17]. However, a relevant problem of metformin therapy are the side effects. Common disturbances are gastrointestinal symptoms, such as abdominal pain, nausea, vomiting, diarrhea, and appetite loss (10–60% of patients) [18]. The side effects are generally of mild intensity and self-limiting. However, these symptoms may reduce the patients’ compliance and cause discontinuation of treatment. There is low evidence to suggest whether one-dose metformin regimen is superior to another. Anyway low doses are preferred to reduce the probability of side effects in clinical practice.

Another class of insulin sensitizers that are commonly used in PCOS treatment nowadays are inositols [19]. Nine stereoisomers of inositols are available, but the most biologically important and widely distributed is myoinositol (MYO). MYO is considered a probiotic molecule and not a real nutrient since it belongs to the vitamin complex but can be synthesized by human cells [20]. Inositols are found in various foods, especially fruit and beans. A small amount is also produced in human cells, even if endogenous production is not sufficient to completely fulfill biological needs [21]. MYO and D-chiro-inositol (DCI) are implicated in glucose homeostasis and insulin signal transduction [22,23,24]. In fact, they show insulin-mimetic properties and contribute to decreasing postprandial blood glucose, acting by different pathways. They both stimulate glucose uptake via Akt activation by up-regulating GLUT4 expression on the plasmatic membrane [24]. Moreover, DCI stimulates glycogen synthesis at the skeletal level [25]. MYO is converted at the cellular level into DCI by an epimerase. It is important to maintain a specific equilibrium between these two isomers to ensure the best control of the insulin signal. Recent studies showed them to be effective in lowering blood pressure, decreasing total and free testosterone, and increasing ovulation frequency by modulating insulin signaling on steroid and ovarian folliculogenesis [24,26,27]. Nevertheless, a Cochrane review [17] found evidence that DCI may improve ovulation rate in PCOS patients, but data regarding the effect on BMI (body mass index), blood pressure, hormonal parameters, except for serum SHBG (sex hormonal binding globulin) levels, fasting glucose and insulin, total cholesterol and triglycerides, were not conclusive. Given the lack of strong evidence, the 2018 international guidelines recommend caution in inositol administration and advise to consider them an experimental therapy for PCOS patients, with emerging evidence on efficacy that requires further studies.

**Figure 1:**
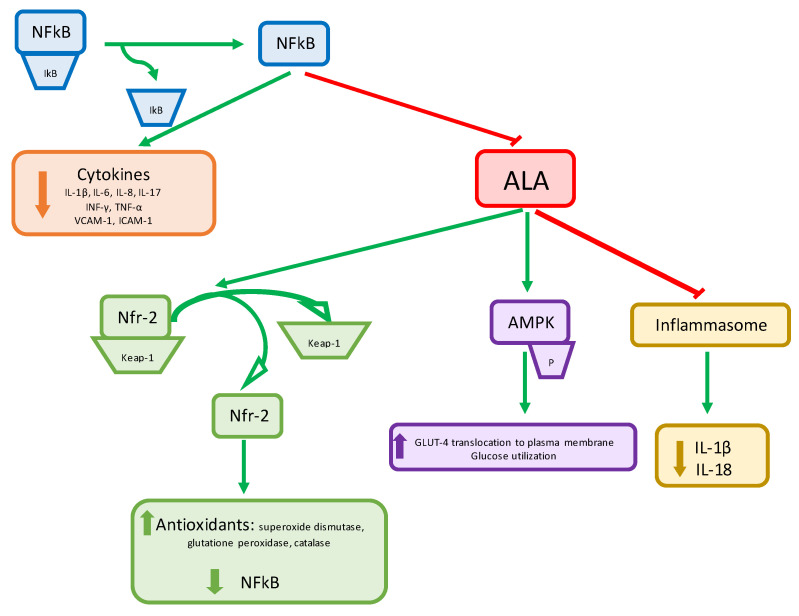
The multiple ALA effects.

Another compound, alpha-lipoic acid (ALA), showed an impact on PCOS treatment in association with inositols. ALA is a natural amphipathic compound produced from octanoic acid in animals and plants with a very strong anti-inflammatory and antioxidant effect [28,29]. It acts along multiple pathways and has many interesting properties, which have been widely described in recent years. First of all, ALA plays a very noteworthy role in the insulin metabolic pathway [30,31,32,33,34] by enhancing glucose uptake. It stimulates the translocation of glucose transporter proteins GLUT4 and GLUT1 via AMPK activation to plasma membrane in adipocytes, mimicking insulin action [34,35], thus increasing insulin sensitivity. Secondly, ALA and dihydro-lipoic acid (DHLA), in their reduced form, are strong antioxidant molecules. They act as scavengers of the reactive oxygen species (ROS) [36] and have an inhibitory effect on the inflammatory pathway mediated by NF-*κ*B, blocking its translocation to the nucleus [37] and reducing proinflammatory cytokine release, playing an anti-inflammatory and immunomodulatory role [38]. Several studies also demonstrated the role of ALA on beta-cell activity. It seems to contrast with the effect of some toxic substances, such as oleic acid or lipopolysaccharide S, which decrease the insulin secretion of beta-cells [39,40]. ALA reduces the production of reactive oxygen species (ROS), limiting oxidative stress and, consequently, cell damage [41]. Some studies evaluated the effect of ALA on beta-cells exposed to IL-1β, which probably is the cytokine responsible for cell injury in type 1 diabetes, but the results were not definitive. If ALA was administered to cells exposed to IL-1β, the insulin secretion returned to normal, but if ALA was administered to cells not exposed to IL-1β, the insulin secretion decreased [42]. In further studies, it was demonstrated that ALA alone decreases insulin secretion but increases insulin sensitivity. This mechanism protects beta-cells from exhaustion, meaning that also ALA alone has a protective effect on these cells [43,44,45]. In this scenario, the modulation of the oxidative balance of beta-cells may have a role also in the prevention of diabetes [46]. The multiple ALA effects are shown in Figure 1. 

Despite its valuable functions, ALA has a short half-life and low bioavailability [35]. These characteristics may represent important limitations for its use for medical purposes [34].

Several studies demonstrated its preventive role in some diseases, such as Alzheimer’s disease, cardiovascular diseases, hypertension, erective dysfunction, cancer, diabetes mellitus [32,33]. Given the multiple effects of ALA, a therapeutic strategy based on the synergy between inositols and ALA could improve insulin resistance, reduce androgen levels, and ameliorate ovulation in PCOS patients [47]. The purpose of our manuscript is to review the principal evidence about the efficacy of PCOS treatment with ALA alone and in association with inositols.

Firstly, it acts as an antioxidant. ALA and its reduced form, dihydro-lipoic acid (DHLA), act as scavengers of reactive oxygen species (ROS) [34] by stimulating the production of antioxidant enzymes, such as glutathione peroxidase and superoxide dismutase 1, inside mitochondria and endoplasmic reticulum via translocation of nuclear factor erythroid-2–related factor 2 (Nfr-2), preventing the decreasing of PPARγ protein [48] and protecting the cell from oxidative damage.

Secondly, it has an inhibitory effect on the inflammatory pathway mediated by NF-*κ*B, blocking its translocation to the nucleus [37] and reducing proinflammatory cytokine release, playing an anti-inflammatory and immunomodulatory role.

Thirdly, several studies demonstrated that ALA plays a very noteworthy role in the insulin metabolic pathway [31,32,33,34,35] by enhancing glucose uptake [36,37,38,47,48,49]. In fact, ALA stimulates the activity of PI3K, and consequently, the phosphorylation of insulin receptor substrate-1 in adipocytes [50]. This process leads to the translocation of GLUT4 to plasma membrane of adipocytes, thus increasing insulin sensitivity [50]. In some hypotheses, this process also leads to an increase in glucose uptake by GLUT4 [51]. ALA also has an effect on AMPK expression in skeletal muscle and in liver [41]. AMPK is linked to fatty acid oxidation and can induce the translocation of GLUT4 [52,53]. In particular, ALA treatment seems to increase AMPK activity in the skeletal muscle and in hepatocytes [54], mimicking some metformin mechanisms [39]. Moreover, the insulin receptor in hepatocytes has a binding site for ALA [55]. In this scenario, ALA can be considered an insulin-mimetic agent [56,57].

Finally, it inhibits the inflammasome and, consequently, downregulates IL-18 and IL-1β levels [49].

## 3. Search Strategy

This study was carried out following the guidelines of the Preferred Reporting Items for Systematic Reviews and Meta-Analyses (PRISMA) [58]. We performed a literature search of PubMed for studies published in English language up to September 2022. We also included the clinical trials in published systematic review articles or in published meta-analysis. We used the key word “α lipoic acid” OR “Alpha-Lipoic Acid” OR “Alpha Lipoic Acid” OR “PCOS” OR “Polycystic ovary syndrome”. Studies were selected with the following inclusion/exclusion criteria: Women affected by PCOS, intervention with MI with or without DCI, assessment of insulin levels, insulin sensitivity, HOMA index, BMI, levels of tryglicerides, hormone levels (testosterone, estradiol, androstenedione, sex hormone-binding globulin plasma, DHEAS, AMH, LH, and FSH), menstrual frequency, oocyte quality; english language. Exclusion criteria were: Duplicate publications, animal or cell culture studies, letters to the editors, case reports. Two researchers reviewed the articles and extracted: Author name, year of publication, country, number of women affected by PCOS, BMI of women affected by PCOS, alpha-lipoic acid and inositol, myo-inositol or D-chiro-inositol dosage, treatment outcomes.

According to this search strategy, we found and analyzed fourteen studies conducted from 2010 to 2022, mainly in Italy. The purpose of this study is to review the existing literature and to evaluate the data showing the efficacy and the limitation of a treatment strategy based on this promising molecule.

## 4. ALA Effects on Metabolic Alterations in PCOS Patients

Most of the studies conducted evaluated the effect of the combinations of MYO plus ALA on the clinical and endocrine features of PCOS. To our knowledge, just one study has investigated the effects of a treatment based on ALA alone in PCOS patients: Masharani et al. administered alpha lipoic acid to six non-obese, nondiabetic patients with PCOS for a cycle of 16 weeks, showing a significant amelioration in insulin sensitivity and a notable decrease in triglyceride levels, demonstrating that ALA also exerts an anti-atherogenic effect [9]. A study conducted by Genazzani et al. [52] demonstrated how the treatment with a combination of alpha lipoic acid (400 mg) plus myo inositol (1 g) for a period of 12 weeks had an effect on improving insulin sensitivity in PCOS obese patients, which had previously resulted hyperinsulinemic after an oral glucose tolerance test. The study had some limitations, such as the lack of a control group (ALA- or *myo*-Ins-only treated patients) and the very small number of patients enrolled. However, these results may suggest that in the complex etiopathogenesis of PCO syndrome, obesity could not be the only trigger for the development of insulin resistance. In fact, it has been hypothesized by Cheang et al. [59] that an anomaly in the intracellular signaling responsible for the production and release of DCI-IGP mediator could be another trigger mechanism at the basis of insulin resistance in PCOS patients. In this group of non-obese patients, the administration of MYO alone is not sufficient to ameliorate compensatory hyperinsulinemia, probably due to an abnormal epimerase activity, which, in turn, results in a reduced DCI-IPG synthesis and release, and not to a nutritional deficiency of inositol. Typically, epimerase activity may be altered in diabetic patients and in PCOS women with a familiar history of diabetes [60]. In this contest, ALA represents a kind of “backup system”. Inositols are introduced with food, whereas ALA is synthesized inside the cells, representing an endogenous and independent mechanism responsible for the final steps of cellular glucose uptake. The molecular mechanism by which ALA administration might reduce insulin resistance in PCOS patients was demonstrated in animal models. The complex mechanisms underlying diabetes type II downregulate the expression of lipoic acid synthase (LASY), an enzyme that is responsible for the ALA synthesis inside the mitochondria [61,62]. The reduction in ALA synthesis inside the mitochondria reduces the amount of glucose uptake at the level of the skeletal muscle cells because ALA regulates glucose utilization stimulating the expression of glucose-transporter-4 (GLUT-4) via AMPK in this tissue, a mechanism believed to be at the basis of insulin resistance. A recent study by Genazzani et al. investigated the effect of the administration of MYO and ALA, alone or in combination, on insulin sensitivity in obese PCOS patients, evaluating the insulin maximal response to an oral glucose tolerance test before and after 12 weeks of integrative treatments. They demonstrated that the HOMA index was improved in all treatment groups, but when MYO was administered alone, it was able to reduce the insulin response only in obese PCOS patients without familial diabetes, where ALA, alone or plus MYO, was instead able to significantly decrease the insulin response, independently of the presence of diabetes. ALA did not change LH levels, enforcing the hypothesis that ALA modulates differently from MYO on different pathways that control LH and GLUT4 activity [54]. A retrospective study by Fruzzetti et al. confirmed these findings. They administered 800 mg of ALA and 2000 mg of MYO per day from 6 to 24 months and showed that the insulin response after an OGTT was significantly reduced, where HOMA-IR was not affected, being already normal in most of the women studied [63]. Another study showed that better results were obtained when ALA was associated with a higher dose of MYO (2000 mg vs. 1000 mg) [45].

These data support the key role of ALA in the modulation of insulin sensitivity and confirm the fact that ALA overcomes the enzymatic impairment induced by familial diabetes due to defects in function and/or mitochondrial LASY synthesis [64].

Besides, evidence from studies conducted on animal and human models suggests that ALA has an effect on the development of adipose tissue and its function by inhibiting adipogenesis, modulating the secretion of many adipokines, such as leptin and apelin, and promoting mitochondrial biogenesis [65,66,67].

Some groups speculated that the treatment based on the combination of MYO plus ALA could improve metabolic features in obese PCOS women through a new mechanism, not completely understood yet, which could be independent from the insulin pathway and centered instead on the ovary [57].

In a recent study by Cirillo et al., they administered a treatment with 400 mg of ALA plus 1000 mg of MYO twice daily for three months, and once daily for further three months in PCOS adolescents and reported a significant reduction in insulin levels and in HMGB1 levels, which is a marker of inflammation, hypothesizing that ALA has a synergistic effect with MYO on lowering HMGB1 and improving insulin sensitivity. However, they did not observe consistent variations in the lipid profile with treatment, at variance with previously reported data in adult women [44].

## 5. ALA Effects on Reproductive Features in PCOS Patients

Various studies investigated the role of ALA in the reproductive function of women affected by PCOS. The beneficial effects of this molecule seem to be related to its anti-inflammatory action and not only to its antioxidant effect. In fact, ALA reduces, on the one hand, the levels of some pro-inflammatory cytokines (TNF-α, IL-1β, IL-6, IL-18, interferon-y) and, on the other hand, the oxidation of glutathione, vitamin C and vitamin E [50]. Moreover, ALA seems to have a role in:

- Hormonal profile
- Menstrual frequency
- Oocyte quality

### 5.1. ALA and Hormonal Profile

This compound seems to have another important function: in some studies, it was demonstrated that the supplementation with ALA influenced hormonal profile. In 2012, Gambera et al. evaluated the effects of supplementation with 600 mg of ALA twice daily for six months in 19 overweight PCOS patients and showed a significant reduction of testosterone levels in treated patients [51]. In 2014, Genazzani et al. treated 34 patients with MYO 1 g and ALA 400 mg for three months and reported a significant reduction of LH levels [55]. At the same time, Iezzi et al. enrolled 26 adolescents affected by PCOS with menstrual irregularities and treated them with 400 mg of inositol and 1000 mg of ALA for six months, obtaining a significant reduction of LH, testosterone, and DHEAS and a significant improvement in estradiol. This study seemed to suggest that ALA could improve ovarian function and oocyte quality by reducing LH and increasing estradiol levels; on the other side, ALA may also reduce hyperandrogenism, decreasing testosterone and DHEAS levels [56].

Moreover, by studying 40 women who received supplementation with 2 g of MYO and 800 mg of ALA, De Cicco et al. found that the hormonal levels were significantly improved after six months, in particular, SHBG, androstenedione, free androgen index (FAI), DHEAS, and AMH [57]. In a more recent study, Genazzani et al. treated 254 overweight/obese PCOS women with 1 g of MYO and 400 mg of ALA, or with MYO or ALA alone for 12 weeks and showed that LH levels were significantly reduced within the group treated with the combined therapy, whereas this result was not found in patients treated with ALA alone. These findings seemed to suggest that it is possible to appreciate the benefits of ALA supplementation [54] when ALA is combined with MYO. In fact, the supplementation with ALA alone did not seem to have a role in improving the hormonal pattern of PCOS patients [52]. The synergistic effect between MYO and ALA can be explained by the hypothesis that the metabolic pattern and insulin secretion influence the signaling pathway involved in ovulation control and in hyperandrogenism. Several studies demonstrated that increased insulin levels may produce a premature expression of LH receptors in small follicles, leading to premature granulosa terminal differentiation and arrest of follicular growth [39,40,48,53]. Other studies hypothesized that insulin may have a direct effect on steroidogenesis. For example, in women affected by PCOS, insulin per se may increase androgen levels (progesterone and androstenedione) and decrease SHBG levels without altering gonadotropin secretion [41,42,43]. 

### 5.2. ALA and Menstrual Frequency

Several studies demonstrated how supplementation with ALA improves menstrual frequency [9,44,45]. In 2015, Cianci et al. screened 62 women and treated them with 1 g of inositol and 600 mg of ALA for 180 days, with an improvement in menstrual cyclicity [46]. In 2019, Fruzzetti et al. demonstrated that the treatment with 600 mg of ALA and 1 g of MYO in 41 women ameliorates the menstrual disturbances. The mean cycle length was 86.8 ± 41 days before the treatment and 41.0 ± 25.4 days after the treatment (*p* < 0.0007) [47].

These studies confirmed that ALA has an important role in improving menstrual cycle length, probably because it restores ovulation in PCOS patients, although it is difficult to establish if the improvement in menstrual cycle was the result of a modulatory effect of the association on gonadotropin secretion and then ovarian steroidogenesis or the consequence of the metabolic changes and BMI reduction [47].

### 5.3. ALA and Oocyte Quality in Patients Who Undergo IVF

It has also been hypothesized that ALA antioxidant function plays a critical role in oocyte quality in PCOS patients. In 2015, Rago et al. analyzed the effect of ALA on oocyte quality in 37 non-obese PCOS patients who underwent in vitro fertilization (IVF). They compared the treatment of at least three months with 2 g of MYO plus 800 mg of ALA versus 4 g of MYO alone and demonstrated that in the first group of patients, the number of germinal vesicles and degenerate oocytes was reduced [68]. Several studies demonstrated that oxidative stress influences apoptosis in post-ovulatory aged oocytes. It was shown that ALA reduced oxidative stress and, consequently, has a role in oocyte quality [69,70,71]. Furthermore, ALA is different from other antioxidants because it is both water-soluble and fat-soluble: this means that this molecule is widely distributed and can elicit its function both in cellular membrane and in cytosol [72]. 

The beneficial effect of ALA combined with MYO in PCOS patients who undergo IVF was also shown by Canosa et al. in 2020. Forty overweight/obese PCOS patients were treated with 2 g of MYO, 800 mg of ALA, and 400 mg of folic acid for three months before IVF. In these patients, the oocytes had a significantly higher inner layer retardance, larger area and thickness of zona pellucida, and a significantly shorter meiotic spindle axis compared to patients treated only with folic acid [73]. These characteristics are associated with better follicular maturation and with a higher developmental competence of the oocyte [74,75]. Moreover, they found a higher proportion of top-quality embryos and an optimal cleavage range. Additionally, the implantation rate resulted higher in patients treated with a combination of MYO and ALA, and consequently, the clinical pregnancy rate and the life birth rate were higher [73]. These studies seemed to confirm the hypothesis that ALA gives an important contribution to oocyte quality in PCOS patients.

The results of the studies reviewed in this paper are summarized in Table 1. 

## 6. Conclusions

Summarizing all the literature evidence analyzed so far, we can conclude that ALA is a valid therapeutic strategy applicable in the treatment of PCOS patients. Its multiple actions, including anti-inflammatory, antioxidant, and insulin-sensitizing, may be of utmost importance in the treatment of a very complex syndrome. Specifically, the combination of MYO plus ALA creates a synergistic effect that improves insulin resistance in PCOS patients, especially in obese/overweight patients with DMT2 familiarity. Of course, not all PCOS women show signs of IR and, to date, accordingly to the last diagnostic criteria, insulin resistance is not included among the distinctive diagnostic features of PCOS. Therefore, this therapeutic effect may be restricted to those PCOS patients with a prevalent metabolic signature.

Nonetheless, ALA treatment also exerts beneficial effects on endocrine patterns, especially if combined with MYO, improving menstrual regularity and ovulation rhythm. There is evidence that inositols are able to impact PCOS hormonal patterns through some pathways that are independent of the metabolic ones, whereas ALA does not seem to have a robust influence on these pathways. Therefore, more data are required to confirm ALA utility in improving inositol activity.

The review has some limitations. First of all, the small number of patients used in the studies. In fact, some studies analyzed only a few patients, for example, Masharani et al. (only 6 patients) or Cirillo et al. (23 patients). Secondly, ALA was generally administered in combination with inositol, therefore, it is difficult to evaluate the real effect of ALA alone. Thirdly, some studies did not report a control group of patients. At last, the majority of these studies was conducted in Italy, so it could be difficult to translate the same therapeutic effect to other populations with different ethnic and racial backgrounds. 

Anyway, according to the observed effects of ALA in all these studies, we may conclude that ALA has a beneficial effect on PCOS patients.

Since the reduced number of studies found in the literature about this topic and the bias of the existing studies, further studies properly conducted, as RCTs or clinical trials enrolling a larger number of patients, are required, in order to establish the effects of the supplementation of ALA alone in PCOS treatment and its efficacy in association with inositols upon the regularization of hormonal patterns in PCOS patients.

## Figures and Tables

**Table 1:** In this table are shown the effects of ALA treatment evaluated by the different research groups. The second last right column shows the treatment regimen administered (ALA alone or in association with DCI or MYO) and the different dosage chosen by the research groups. The last column shows the results of the treatment. (↓: reduction; ↑: increase).

Author	Year	Country	PCOS (Number)	BMI PCOS	Treatment	Results
Masharani [9]	2010	USA	6	>25	ALA 600 mg twice daily	- ↓ levels of trygliceride - ↑ insulin sensitivity - ↑ menstrual frequency
Gambera [51]	2012	Italy	19	>25	ALA 600 mg twice daily	- ↓ testosterone levels
Genazzani [55]	2014	Italy	34	>25	ALA 400 mg/day + Myo-Ins 1 g/day	- ↓ LH levels - ↑ HOMA index in hyperinsulinemic PCOS
Rago [68]	2015	Italy	37	<25	ALA 800 mg/day + Myo-Ins 2 g/day	- ↓ insulin levels - ↓ BMI - ↑ oocyte quality
Cianci [46]	2015	Italy	46	>25	ALA 600 mg/day + DCI 1 g/day	- ↑ menstrual frequency
De Cicco [57]	2017	Italy	40	>25	ALA 800 mg/day + Myo-Ins 2 g/day	- ↑ menstrual frequency - ↑ hormonal milieu (SHBG, androstenedione, free androgen index, DHEAS, AMH) - ↓ BMI
Genazzani [52]	2018	Italy	32	>25	ALA 400 mg/day	- ↓ HOMA index and BMI
Genazzani [54]	2019	Italy	76	>25	24 patients: Myo-Ins 1 g/day; 24 patients: ALA 400 mg/day; 28 patients: Myo-Ins 1 g/day + ALA 400 mg/day	- ↓ insulin levels - ↓ LH levels - ↑ menstrual frequency
Fruzzetti [47]	2019	Italy	41	>25	ALA 600 mg/day + DCI 1 g/day	- ↑ menstrual frequency
Cirillo [44]	2020	Italy	23	>25	ALA 400 mg/twice daily + MYO 1 g/twice daily (for 3 months, then daily for 3 months)	- ↓ insulin levels
Fruzzetti [63]	2020	Italy	44	>25	ALA 800 mg/day + Myo-Ins 2 g/day	- ↓ BMI - ↑ menstrual frequency
Canosa [73]	2020	Italy	40	>25	ALA 800 mg/day + MYO 2 g/day	- ↑ oocyte quality
Fruzzetti [45]	2020	Italy	71	>25	43 patients: ALA 800 mg/day + Myo-Ins 2 g/day; 28 patients: ALA 800 mg/day + Myo-Ins 1 g/day	- ↓ BMI - ↑ gonadotropin secretion
Iezzi [56]	2022	Italy	26	<25	ALA 1000 mg/day + inositol 400 mg/day	- ↓ LH levels, testosterone and DHEAS - ↑ estradiol levels

## Data Availability

Not applicable.

## Abbreviations

ALA	Alpha lipoic acid
AMH	Anti-müllerian hormone
BMI	Body mass index
DCI	D-chiro-inositol
DHEAS	Dehydroepiandrosterone sulfate
DHLA	dihydro-lipoic acid
DM	Diabetes mellitus
IR	Insulin resistance
LH	Luteinizing hormone
MYO	Myoinositol
FSH	Follicle-stimulating hormone
PCOS	Polycystic ovary syndrome
RCTs	Randomized control trials
ROS	Reactive oxygen species
